# An Iron-NDC Framework with a Cage Structure and an Optothermal Conversion in NIR Window

**DOI:** 10.3390/molecules27248789

**Published:** 2022-12-11

**Authors:** Bin Tan, Zhao-Feng Wu, Xiao-Ying Huang

**Affiliations:** 1 State Key Laboratory of Structural Chemistry, Fujian Institute of Research on the Structure of Matter, Chinese Academy of Sciences, Fuzhou 350002, China; 2 Fujian Science & Technology Innovation Laboratory for Optoelectronic Information of China, Fuzhou 350108, China

**Keywords:** metal-organic framework, cage structure, optothermal effect, NIR laser light

## Abstract

Pursuing novel materials with efficient photothermal conversion under irradiation at the near-infrared region windows (NIR, 750–850 nm; NIR-I and NIR-II, 1000–1320 nm)) is of great importance due to their irreplaceable applications, especially in the biomedical field. Herein, on the basis of a coordination chemistry strategy, an iron-based metal-organic framework (MOF) of [N(CH_3_)_4_]_2_[Fe_3_(NDC)_4_]·DMF·3H_2_O (**Fe-NDC**, 1,4-H_2_NDC = 1,4-naphthalenedicarboxylic acid, N(CH_3_)_4_^+^ = tetramethyl-ammonium, and DMF = N,N-dimethylformamide) was prepared and characterized. Due to the *d*-*d* transition effect introduced by coordination with the transition-metal ion of iron and the highly conjugated naphthalenic moiety in 1,4-H_2_NDC, guaranteeing an energy transfer between iron and the organic module, **Fe-NDC** shows a remarkable broad absorption, which could be extended into the NIR-II section. As a result, **Fe-NDC** could be irradiated by NIR laser (both 808 and 1064 nm) to achieve photothermal conversion. This work sets a good example to inspire the future designation of NIR light-irradiated photothermal materials based on the first-row transition metals.

## 1. Introduction

Photothermal materials have attracted considerable attention in recent years due to their attractive applications in water desalination, thermal therapy, night vision sensors, etc. [1,2,3,4]. With respect to actual applications, such materials are expected to have a strong absorption in the near-infrared region (NIR). Considerable progress has been made in the development of conventional NIR photothermal materials for inorganic and organic semiconductors, such as transition-metal-containing chalcogenides, carbon complexes, polyaniline, and polypyrrole [5,6,7,8,9]. Denoted as the second near-infrared window (NIR-II), light sources beyond 1000 nm are popular in biomedical applications [10,11,12,13]. However, photothermal materials with efficient absorption in the NIR-II window are still limited. The exploration of novel photothermal materials, especially those irradiated by NIR-II light, remains a challenging task.

In recent decades, metal-organic frameworks (MOFs) have been well developed due to their aesthetic structures and varied aspects of applications [14,15,16,17]. Taking advantage of arbitrarily designable ligands and abundant metal resources, MOFs have been explored as promising photothermal materials [18,19]. Examples of such materials that have been investigated include but are not limited to HKUST-1, UiO-66-NH_2_, and ZIF-67 [20]. As for designation of efficient photothermal MOFs, organic ligands are taken into account first. Ligands containing tetrathiafulvalene (TTF), perylene diimide, and viologen moieties are preferred for direct assembly of MOFs or for incorporation into MOFs to fabricate host-guest materials with absorption bands in the NIR region [21,22,23,24]. However, most such photothermal MOFs reported to date have only shown light absorption in the NIR-I window. Transition-metal ions, e.g., copper and iron ions, have been well explored to assemble coordination complexes with catalytic and magnetic properties. On the basis of a coordination chemistry strategy, *d*-*d* transition could extend the absorption of transition-metal-bearing compounds into the visible region [25,26,27,28]. As for MOFs, during the self-assembly process, efficient energy transfers between the conjugated organic ligands and transition metals could further endow the resultant MOFs with broad absorption, offering an additional opportunity to achieve absorption in the NIR region.

Herein, by utilizing 1,4-NDCH_2_ with naphthalene as a good light-absorbing moiety to assemble with iron ions, an iron-based MOF formulated as [N(CH_3_)_4_]_2_[Fe_3_(NDC)_4_]·DMF·3H_2_O (**Fe-NDC**, 1,4-H_2_NDC = 1,4-naphthalenedicarboxylic acid, N(CH_3_)_4_^+^ = tetramethyl-ammonium, DMF = N,N-dimethylformamide) is presented. **Fe-NDC** features a three-dimensional (3D) framework with two types of cavities. **Fe-NDC** exhibits an impressive board absorption band from 300 to 1500 nm and can therefore be heated to 135 °C within seconds under 1064 nm NIR laser at a power density of 1.25 W/cm^2^, demonstrating a remarkable photothermal effect in the NIR-II window and making it a promising NIR-II photothermal material.

## 2. Results and Discussions

### 2.1. Crystal Structure Description

**Figure 1:**
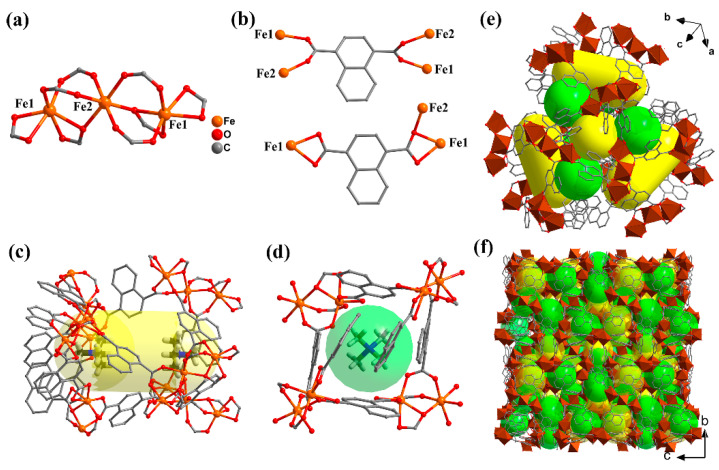
(**a**) The coordination environment for iron ions. (**b**) The two coordination modes of 1,4-NDCH_2_ in **Fe-NDC**. (**c**) and (**d**) Structural diagrams showing the type 1 and type 2 cages, respectively. The cavities are highlighted as yellow and green balls. The tetramethyl-ammonium cations occupy the cavities of these cages. (e) and (**f**) The 3D structures of **Fe-NDC** viewed from different directions. Hydrogen atoms and ammonium cations are omitted for clarity.

**Fe-NDC** was synthesized by a solvothermal reaction with ferrocene, 1,4-NDCH_2_ in a mixture with DMF and CH_3_OH solvents (Figure S1). Single-crystal X-ray diffraction analyses revealed that **Fe-NDC** features a 3D framework comprising iron ions and 1,4-NDC^2−^ ligands. As depicted in Figure 1a, there are two crystallography independent sites for iron ions. Fe(1) is *hex*-coordinated with two carboxylic groups adopting a chelating coordination mode, and the last two coordinated sites are occupied by two COO^−^ groups via monodentate coordination. Fe(2) is coordinated with six monodentate COO^−^ groups, acting as a center to connect two Fe(1) atoms to form a linear trinuclear cluster. The linear trinuclear iron clusters of {Fe_3_(COO)_8_}*_n_*, as the secondary building blocks (SBUs), are further bridged by 1,4-NDC^2−^ ligands in L1 and L2 coordination modes (Figure 1b) to generate a 3D framework. There are two types of cages in **Fe-NDC**. As shown in Figure 1c, in **Fe-NDC**, six {Fe_3_(COO)_8_} units are linked by six L1 and three L2 ligands to form a cylindrical cage (cage 1, Figure S2). Cage 2 is constructed from four SBUs bridged by two L1 and four L2 linkers (Figure 1d and Figure S3). Each cage (1) is surrounded by three of the neighboring cages of the same type (1) and six different cages (2) to generate the resultant 3D structure (Figure 1e,f, Figures S4 and S5). The solvent guests and the tetramethyl-ammonium cations generated in situ [29,30,31] act as charge balance agents and occupy the cavities of the cages in **Fe-NDC** (Figure 1c,d). According to the charge balance and XPS analysis (Figure S5), the valence state of iron is divalent.

### 2.2. Basic Physical Measurements

**Figure 2:**
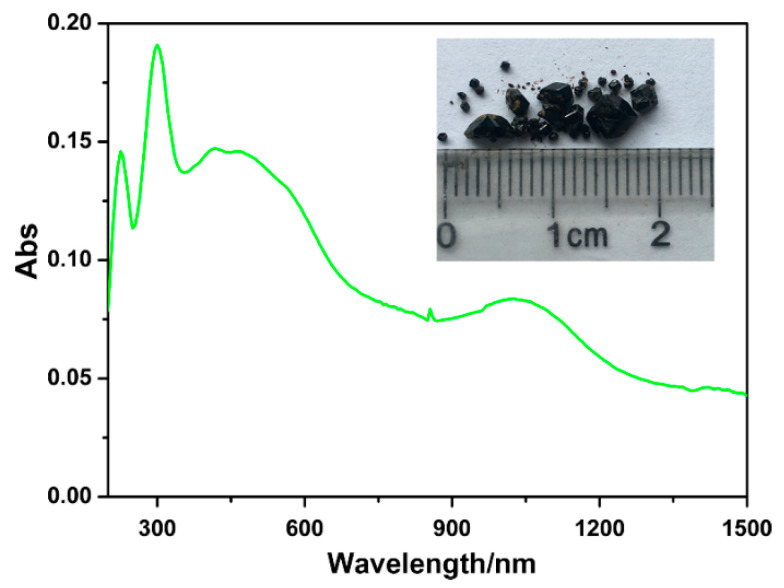
UV-Vis spectra of **Fe-NDC** in solid state at room temperature. The inset is a photograph of black **Fe-NDC** crystals under daylight.

The phase purity of **Fe-NDC** was confirmed by powder X-ray diffraction (PXRD) measurements by comparing the experimental pattern with that calculated based on single-crystal X-ray diffraction data (Figure S6). Thermogravimetric analysis and the PXRD measurements of the title compound immersed under commonly lab-used solvents indicate that **Fe-NDC** has good thermal and antisolvent stabilities (Figures S7 and S8). **Fe-NDC** shows a black prismatic-like morphology in the millimeter size range, which has an efficient light absorption capacity, as further demonstrated by the solid UV-Vis diffuse reflectance spectra (inset of Figure 2). As shown in Figure 2, **Fe-NDC** exhibits a long-wavelength window of absorption ranging from 300 to 1500 nm. Compared with the intense absorption peak around 400 nm for the free 1,4-NDCH_2_ ligand, the remarkable long-wavelength absorption of **Fe-NDC** was attributed to the *d*-*d* transition of iron ions through coordination with 1,4-NDC^2−^ ligands [25,26,27,28].

### 2.3. Photothermal Conversion Characterizations

**Figure 3:**
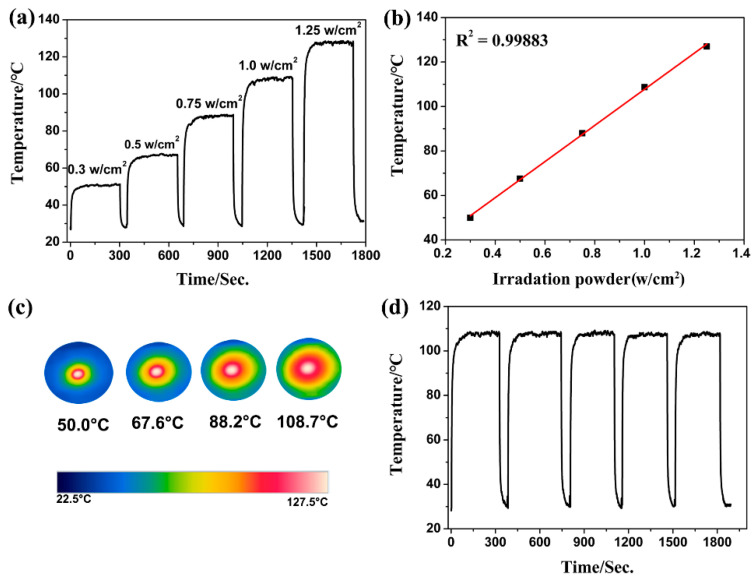
(**a**) Photothermal conversion curves of **Fe-NDC** under 808 nm laser irradiation from 0.30 to 1.25 w/cm^2^. (**b**) The linear relationship between the energy powder and temperature. (**c**) Photographs of **Fe-NDC** with different irradiation powders monitored by an infrared thermal imager. (**d**) Photothermal cycling curve for **Fe-NDC** under 1.00 W/cm^2^ irradiation.

The remarkable near-infrared absorption of **Fe-NDC** suggests an efficient conversion of infrared light into thermal energy. Therefore, the photothermal properties of **Fe-NDC** as a NIR photothermic MOF were investigated in detail. As depicted in Figure 3, under the irradiation of an 808 nm NIR laser, the surface temperature of the powdered **Fe-NDC** rapidly increased to 50.0 °C from room temperature within seconds, with a power density of 0.30 w/cm^2^, reaching 127.5 °C at 1.25 w/cm^2^ (Figure 3a–c). This performance is better than that of most reported MOF-based materials irradiated under UV light or 808 nm irradiation (Table S2) [20,32,33,34,35,36]. The photothermal behavior of **Fe-NDC** exhibits a positive linear relationship with laser power, indicating laser irradiation powder-dependent optothermal conversion performance (Figure 3b). The title compound also exhibited no performance decay after five cycles of experiments under 1.00 W/cm^2^ irradiation (Figure 3d), demonstrating its photothermal stability and durability, even at relatively high temperatures. The isostructural **Co-NDC** with similar chemical tolerance was also synthesized for comparative study of the photothermal conversion performances (Figures S9–S11). As shown in Figures S12–S14, **Fe-NDC** shows better light-to-heat transfer ability than **Co-NDC** under the same measuring conditions, demonstrating that iron is a comparatively appropriate candidate to construct photothermal MOFs under NIR.

**Figure 4:**
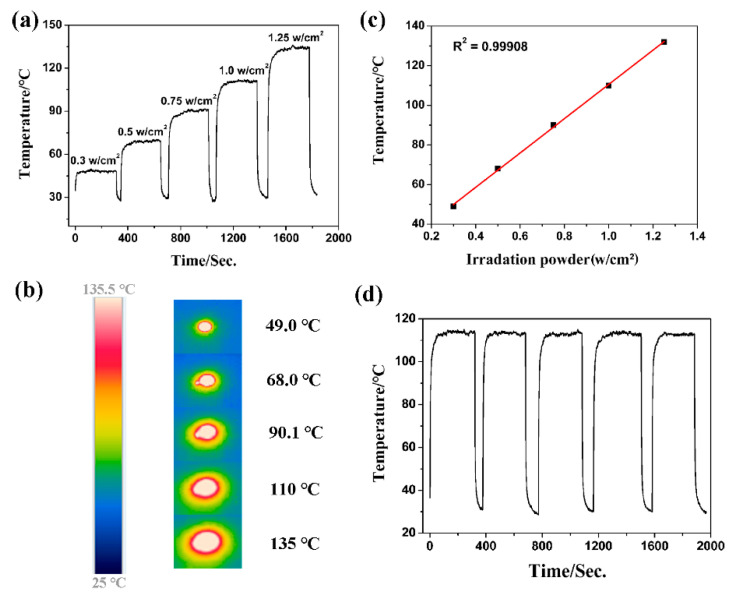
(**a**) Photothermal conversion curves of **Fe-NDC** under 1064 nm laser irradiation from 0.30 to 1.25 w/cm^2^. (**b**) The linear relationship between the energy powder and temperature. (**c**) Photographs of **Fe-NDC** with different irradiation powders monitored by an infrared thermal imager. (**d**) Photothermal cycling curve of **Fe-NDC** under 1064 nm laser at 1.00 W/cm^2^ power density.

**Figure 5:**
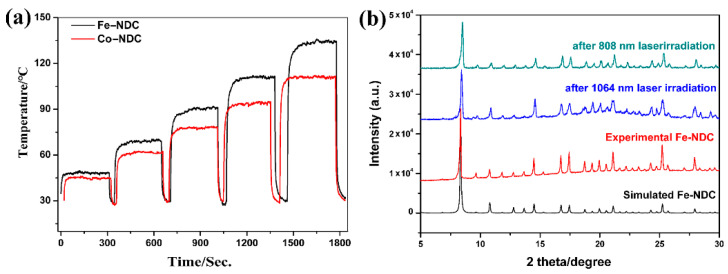
(**a**) Photothermal conversion curves of **Fe-NDC** under 1064 nm laser irradiation from 0.30 to 1.25 w/cm^2^. (**b**) Experimental PXRD patterns of **Fe-NDC** after cycling irradiation tests with 808 and 1064 nm lasers compared with the simulated pattern.

Photothermal materials that can work in the NIR-II region are highly desirable, especially for biomedical applications. To the best of our knowledge, MOFs with an optothermal effect in the NIR-II region are still rare [37]. **Fe-NDC** also exhibits an obvious absorption maximized at 1100 nm, suggesting that efficient optothermal conversion might be generated in the NIR-II window. As anticipated, **Fe-NDC** exhibited a satisfactory photothermal performance under 1064 nm laser irradiation. The heating curves of **Fe-NDC** with irradiation at different power densities are shown in Figure 4a; the temperature increased to 49.0, 68.0, 90.1, 110.0, and 135.0 °C within seconds under 1064 nm irradiation with 0.25, 0.50, 0.75, 1.00, and 1.25 W/cm^2^, respectively (Figure 4b). A positive linear relationship between the temperature increment and the NIR laser power from 0.25 to 1.25 W·cm^−2^ indicates a good thermal control performance of **Fe-NDC** (Figure 4c). As shown in Figure 4d, no substantial deterioration of the photothermal performance was observed after at least six cycles of irradiation. Although the isostructural **Co-NDC** also exhibited good photothermal conversion performance under the same conditions, the resultant temperature was lower than that of **Fe-NDC** (Figure 5a and Figures S14–S16). In the UV-Vis absorption of **Co-NDC** (Figure S17), the absorption intensities at 808 nm and 1064 nm were much lower than those of **Fe-NDC** (Figure 2), which could explain the low photothermal conversion. PXRD of the sample after the 1064 nm laser cycling irradiation tests was also measured, in agreement with the simulated pattern, indicating the photostability of **Fe-NDC** (Figure 5b). The light-to-heat conversion performance of **Fe-NDC** is better than that of most reported MOF-based materials, even under UV or NIR-I light irradiation [34,37] (Table S1). These results suggest that **Fe-NDC** is a promising candidate optothermal material for the NIR-II window.

## 3. Materials and Methods

**Synthesis of the compounds**. All reagents and chemicals were purchased from commercial sources and used without further purification.

**Synthesis of Fe-NDC**: A mixture of ferrocene (0.5 mmol, 0.093 g) and 1,4-NDCH_2_ (1 mmol, 0.216 g) with 4 mL DMF and 1 mL CH_3_OH was sealed in an autoclave equipped with a 20 mL Teflon-lined bomb and heated to 160 °C for 4 days and then cooled to room temperature. Black crystals were obtained by filtration and ethanol washing. Anal. Calc. for **1**: C 54.52%, H 4.73%, N 3.23%. Found: C 54.87%, H 4.56%, N 3.20%.

**Synthesis of Co-NDC**: The synthesis procedure was the same as that for the **preparation of Fe-NDC**, replacing ferrocene with Co(NO_3_)_2_·6H_2_O. Purple crystals were obtained by filtration and ethanol washing.

**Physical measurements**. Powder X-ray diffraction (PXRD) patterns were recorded on a Rigaku MiniFlex II diffractometer using CuK*α* radiation (*λ* = 1.5406 Å). Graphite monochromator was used, and the generator power settings were set at 44 kV and 40 mA. Data were collected between 2*θ* of 3 and 50° with a scanning speed of 1.0°/min. Thermogravimetric (TG) data were collected on a TA Q50 analyzer with a temperature ramping rate of 10 °C/min from 30 to 700 °C under nitrogen gas flow. Elemental analyses for C, H, and O were performed on a German Elementary Vario EL III instrument. UV-Vis diffuse reflectance spectra were measured at room temperature using a PE Lambda 950 UV-Vis spectrophotometer. The spectrophotometer was calibrated against the surface of BaSO_4_ for 100% reflectance over the wavelength range under consideration for UV-Vis diffuse reflectance spectra measurements. Single-crystal X-ray diffraction data were collected with graphite-monochromated MoK*α* (*λ* = 0.71073 Å) using an XcaliburE CCD diffractometer at 100 K.

**Photothermal Experiments**. The powdered sample (10 mg) was spread on quartz slides to form thin, round layers at a fixed height from the light guide. During the stepwise photothermal test, the powdered samples keep immovable. An 808 or 1064 nm laser was generated with an infrared diode laser (MDL-III-800-10 W from Changchun New Industries Optoelectronics Tech Co. Ltd., Jilin, Changchun 130103, China) and irradiated at different laser power intensities. An infrared thermal imager (FOTRIC 326C) was used to capture infrared videos of the samples. Infrared photos and real-time temperatures for the samples were extracted from the video. To evaluate the photothermal stabilities, the samples were irradiated with a laser at a defined power intensity for 5 min and then naturally cooled to room temperature. The on/off cycle was repeated five times.

**X-ray crystallography**. A single crystal of **Fe-NDC** or **Co-NDC** suitable for single-crystal X-ray diffraction (SCXRD) was selected under an optical microscope and glued to a thin glass fiber. The structures were solved by direct methods and refined with full-matrix least squares techniques using the *SHELX*2018 package [38]. The CCDC numbers for **Fe-NDC** and **Co-NDC** are 2214948 and 2222741, respectively. Detailed crystallographic data and structure-refinement parameters are summarized in Table 1.

## 4. Conclusions

In summary, a transition-metal-based MOF assembled from iron and 1,4-NDCH_2_ was presented and characterized. The as-made **Fe-NDC** shows a broad wavelength window of absorption that extends into the infrared region, resulting in an efficient optothermal effect under NIR laser irradiation both at 808 and 1064 nm. Remarkably, **Fe-NDC** can be quickly heated up to 135 °C within seconds under 1064 nm laser, with a power density of 1.25 W/cm^2^. The NIR-II light-driven photothermal converting performance and good chemical tolerance make **Fe-NDC** a promising material for applications in water purification, photothermal therapy, thermal catalysis, etc. Additional MOFs with optothermal applications will be designed and synthesized in our lab in the future.

## Figures and Tables

**Table 1:** Crystallographic data and structural refinement details for **Fe-NDC** and **Co-NDC**.

Empirical Formula	C_59_H_61_Fe_3_N_3_O_20_	C_59_H_61_Co_3_N_3_O_20_
Formula weight	1299.65	1308.89
Crystal system	Cubic	Cubic
Space group	*I*2_1_3	*I*2_1_3
T/K	100(2)	295(2) K
*λ*/Å	0.71073	0.71073
*a*/Å	25.80060(10)	25.6346(3)
*b*/Å	25.80060(10)	25.6346(3)
*c*/Å	25.80060(10)	25.6346(3)
*α*/º	90	90
*β*/º	90	90
*γ*/º	90	90
*V*/Å^3^	17,174.7(2)	16,845.3(6)
*Z*	12	12
*D_c_*/Mg·m^−3^	1.508	1.548
*μ*/mm^−1^	0.831	0.959
*F*(000)	8088	8124
Measured refls.	89,269	12,550
Independent refls.	7772	6278
*R*_int_	0.0398	0.0249
No. of parameters	362	383
*GOF*	1.048	1.067
*^a^ R*_1_, *^b^ wR*_2_ [*I* > 2*σ*(*I*)]	0.0517, 0.1410	0.0441, 0.1255
*^a^ R*_1_, *^b^ wR*_2_ (all data)	0.0613, 0.1525	0.0596, 0.1390

*^a^ R*_1_ = ∑║*F*_o_│ − │*F*_c_║/∑│*F*_o_│. *^b^ wR*_2_ = [∑*w*(*F*_o_^2^ӱ *F*_c_^2^)^2^/∑*w*(*F*_o_^2^)^2^]^1/2^.

## Data Availability

All the available data are incorporated in the MS.

## Supplementary Materials

The following supporting information can be downloaded at: https://www.mdpi.com/article/10.3390/molecules27248789/s1. Figure S1: The image of crystals of **Fe-NDC**; Figure S2: The structures for cage 1 and cage 2 in **Fe-NDC**; Figure S3: The structures for cage 1 surrounded with the same three neighboring cages in **Fe-NDC**; Figure S4: The 3D structures of **Fe-NDC** constructed with cages 1 and cages 2; Figure S5: The XPS spectra of Fe (2p) in **Fe-NDC**; Figure S6: Experimental PXRD patterns of **Fe-NDC** compared with the simulated one. Figure S7: TG curve for **Fe-NDC**; Figure S8: Experimental PXRD patterns of **Fe-NDC** under different solvents over 48 h compared with the simulated **Fe-NDC**; **Figure S9**: Experimental PXRD patterns of **Co-NDC** compared with the simulated **Co-NDC**; Figure S10: IR spectra of 1,4-NDCH_2_ ligand, **Fe-NDC** and **Co-NDC**; Figure S11: Experimental PXRD patterns of **Co-NDC** under different solvents over 48 h compared with the simulated **Co-NDC**; Figures S12 and S15: Photothermal conversion curves of **Co-NDC** under 808 and 1064 nm laser irradiation from 0.30 to 1.25 w/cm^2^; Figures S13 and S16: Photothermal cycling curve of the **Co-NDC** at 1.25 W/cm^2^ irradiation under 808 nm and 1064 nm laser light; Figure S14: Photothermal conversion curves of **Fe-NDC** and **Co-NDC** under 808 nm laser irradiation from 0.30 to 1.25 w/cm^2^; **Figure S17**: Vis spectra of **Co-NDC** in solid state measured at room temperature with the photograph of the as-made **Co-NDC** as inset; Table S1: Summary of the photothermal conversion efficiency of various MOF involved photothermal agents. References [39] are cited in the Supplementary Materials.
