# Supplementary material for: An Iron-NDC Framework with a Cage Structure and an Optothermal Conversion in NIR Window
Source: Molecules. 2022 Dec 11;27(24):8789. doi: 10.3390/molecules27248789 (PMC9785624; doi:10.3390/molecules27248789)
Supplement: Supplementary file 1 [file molecules-27-08789-s001.zip › molecules-2019700-supplementary.pdf]

An iron-NDC framework with a cage structure and an optothermal conversion in NIR window

Bin Tan [1,2], Zhao-Feng Wu [1,2,*] and Xiao-Ying Huang [1,*]

[1] State Key Laboratory of Structural Chemistry, Fujian Institute of Research on the Structure of Matter, the Chinese Academy of Sciences, Fujian, Fuzhou, 350002, P.R. China

[2] Fujian Science & Technology Innovation Laboratory for Optoelectronic Information of China, Fujian, Fuzhou 350108, P. R. China

* Correspondence: zfwu@fjirsm.ac.cn (Z.-F. Wu); xyhuang@fjirsm.ac.cn (X.-Y. Huang); Tel.: 0591-63173146 (X.-Y. Huang).

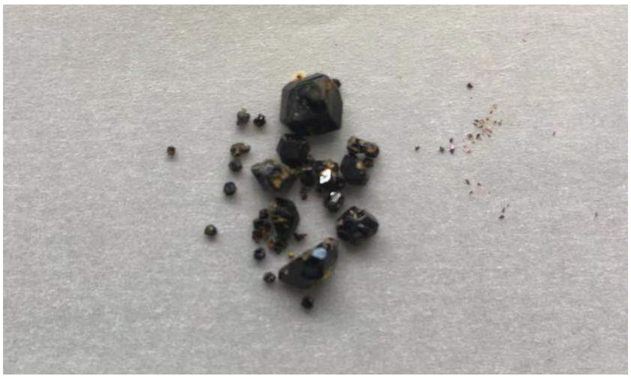

Figure S1. The image of crystals of **Fe-NDC**.

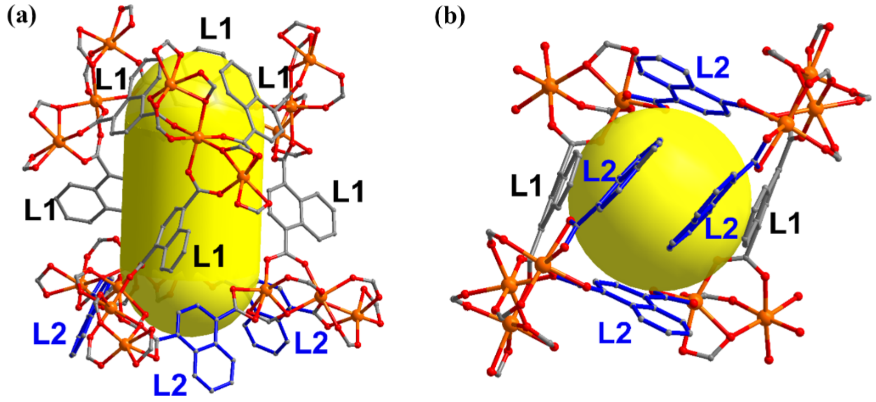

Figure S2. The structures for cage 1 and cage 2.

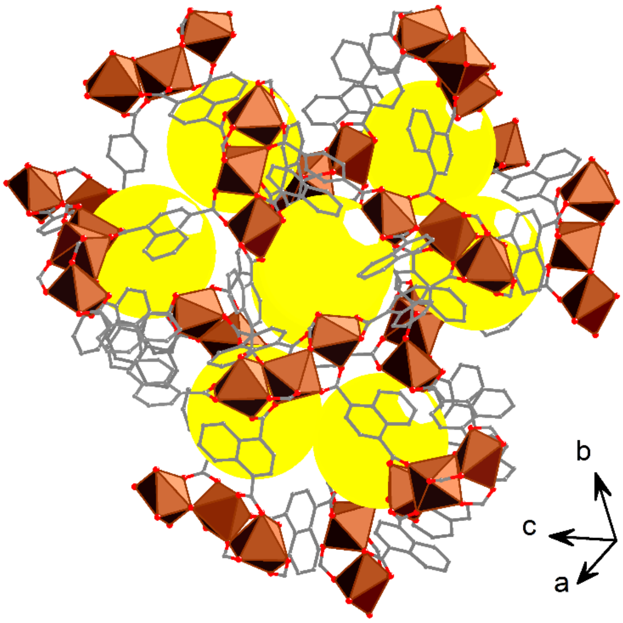

Figure S3. The structures for cage 1 surrounded with the same three neighboring cages.

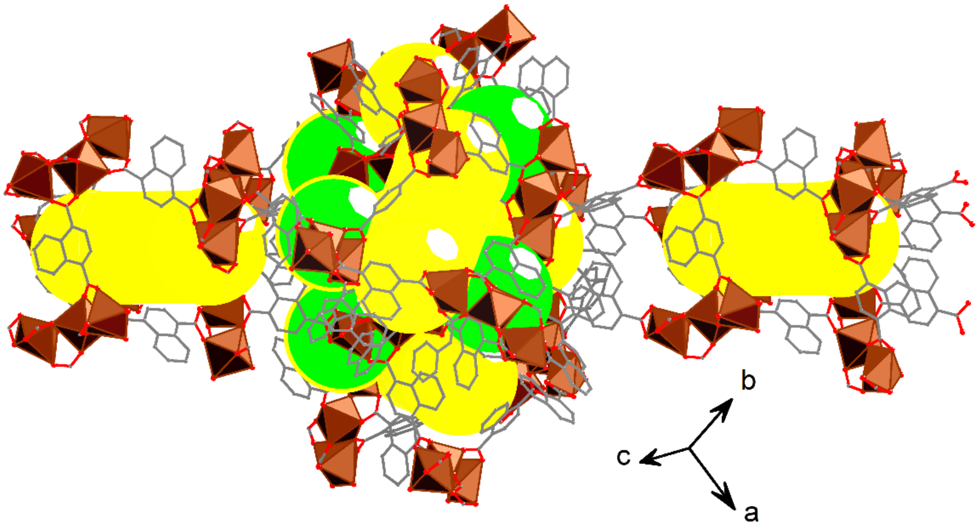

Figure S4. The 3D structures of **Fe-NDC** constructed with cages 1 and cages 2.

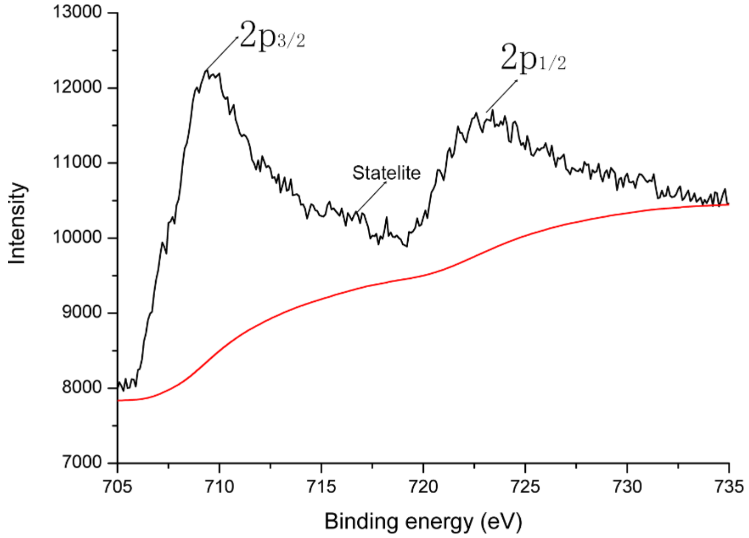

Figure S5. The XPS spectra of Fe (2p) in **Fe-NDC**.[S1]

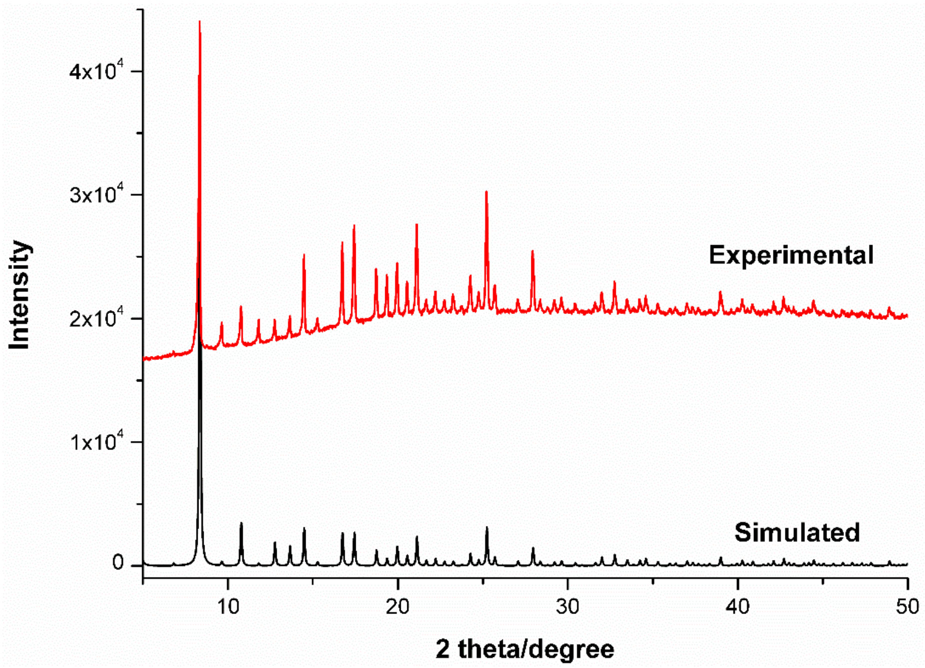

Figure S6. Experimental PXRD patterns of **Fe-NDC** compared with the simulated one.

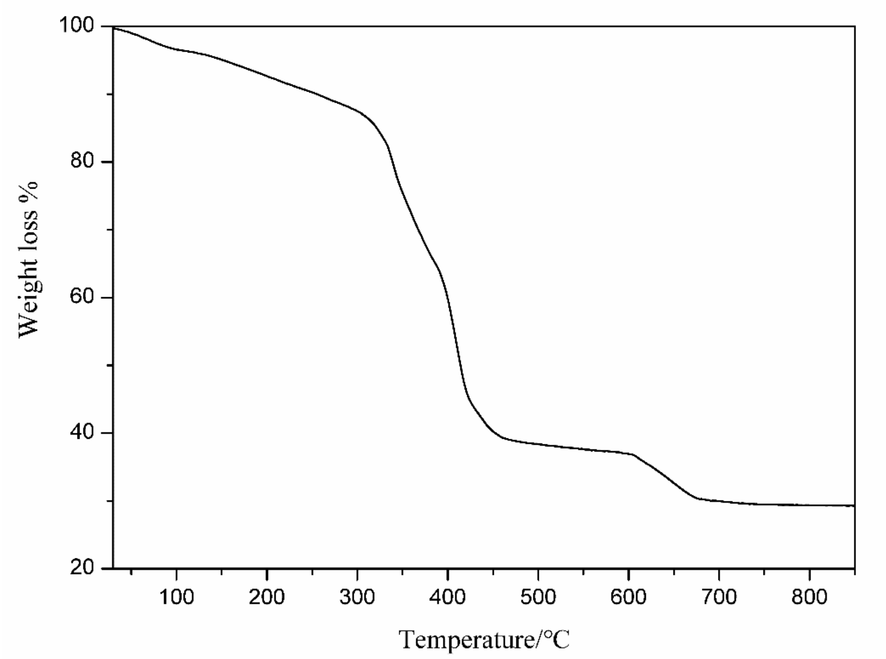

Figure S7. TG curve for **Fe-NDC**.

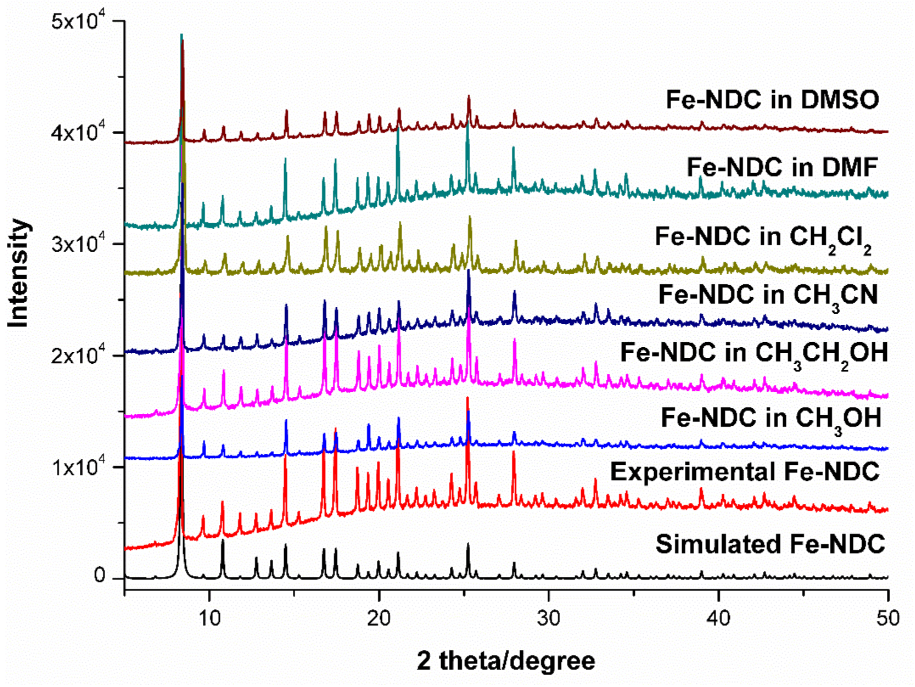

Figure S8. Experimental PXRD patterns of **Fe-NDC** under different solvents over 48 hours compared with the simulated **Fe-NDC**.

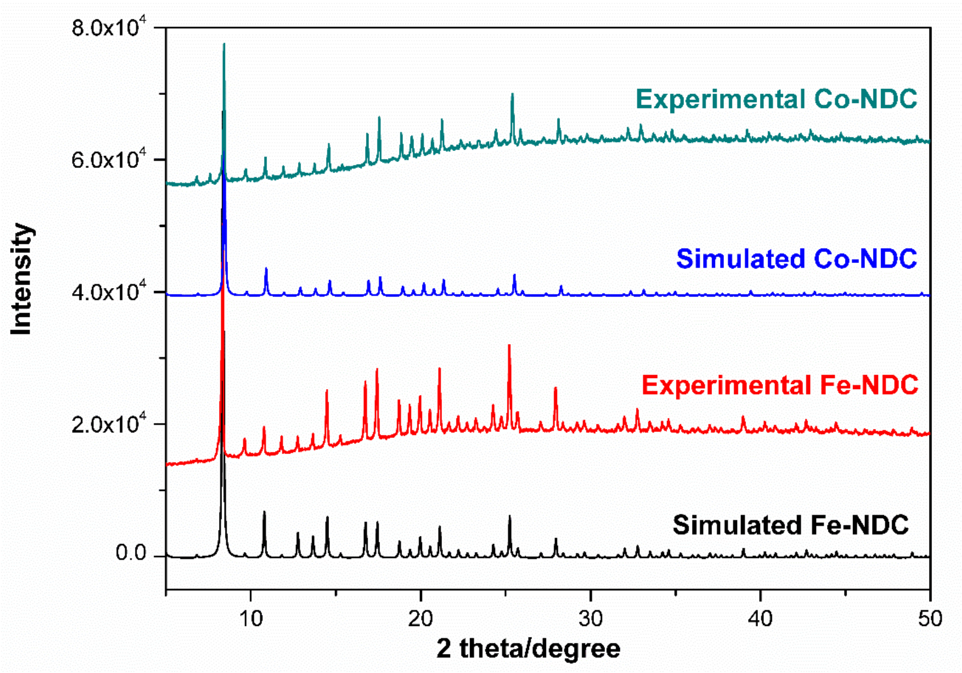

Figure S9. Experimental PXRD patterns of the isostructural **Co-NDC** compared with the simulated **Co-NDC**. The PXRD patterns of Fe-NDC are added for comparatively study.

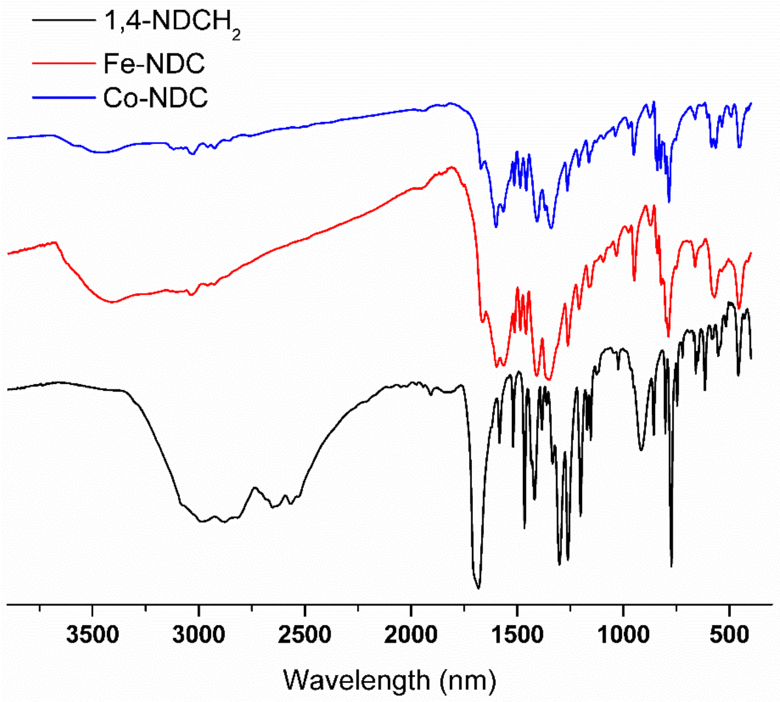

Figure S10. IR spectra of 1,4-NDCH2 ligand, **Fe-NDC** and the isostructural **Co-NDC**.

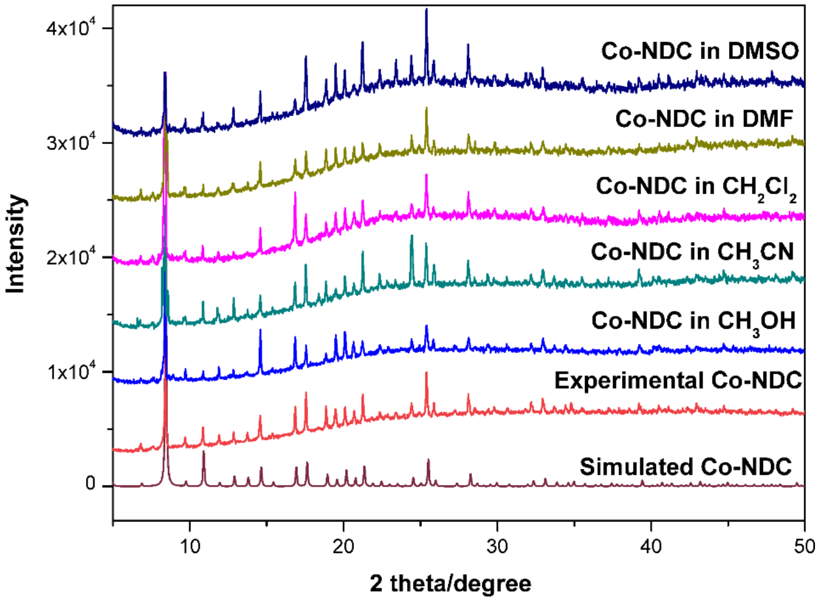

Figure S11. Experimental PXRD patterns of **Co-NDC** under different solvents over 48 hours compared with the simulated **Co-NDC**.

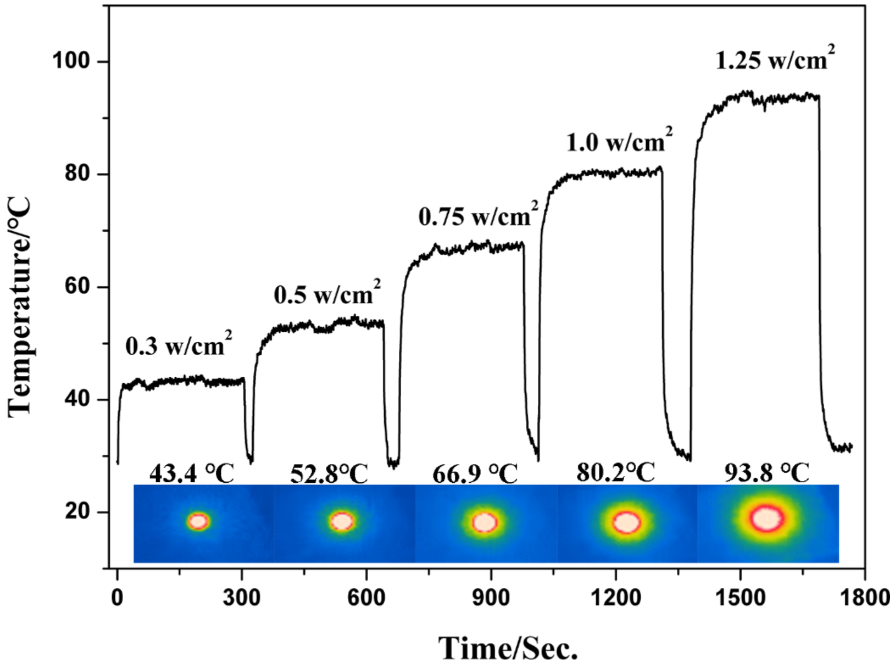

Figure S12. Photothermal conversion curves of **Co-NDC** under 808 nm laser irradiation from 0.30 to 1.25 w/cm^2. Inset is the photographs of **Co-NDC** at different irradiation powder monitored by the infrared thermal imager.

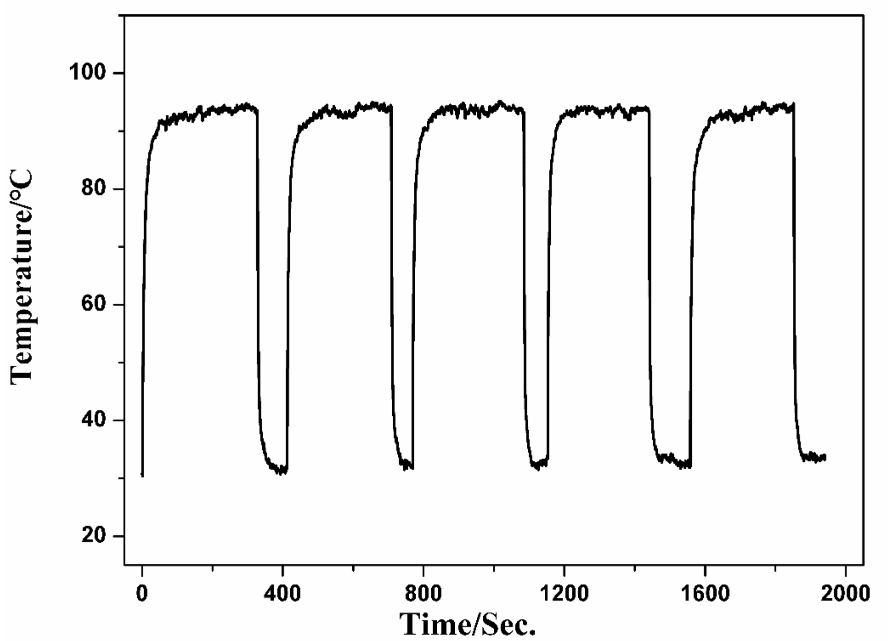

Figure S13. Photothermal cycling curve of the **Co-NDC** at 1.25 W/cm^2 irradiation (808 nm).

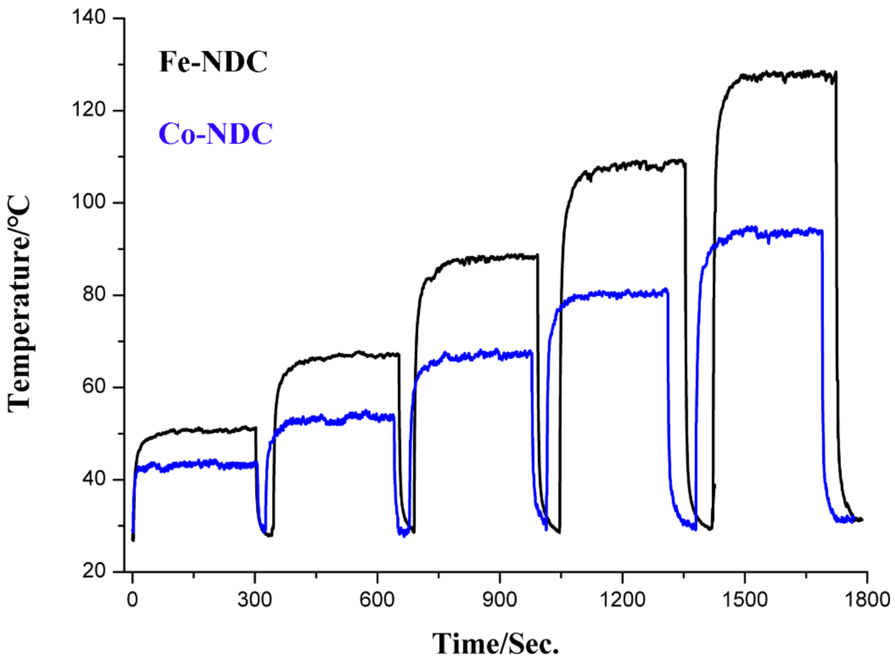

Figure S14. Photothermal conversion curves of **Fe-NDC** and **Co-NDC** under 808 nm laser irradiation from 0.30 to 1.25 w/cm^2.

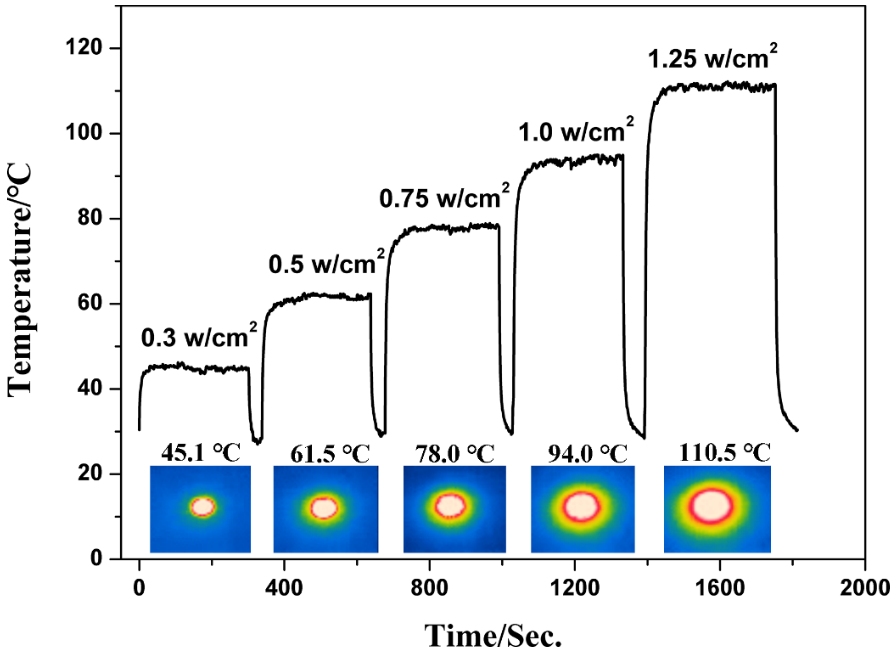

Figure S15. Photothermal conversion curves of **Co-NDC** under 1064 nm laser irradiation from 0.30 to 1.25 w/cm^2. Inset is the photographs of Co-NDC at different irradiation powder monitored by the infrared thermal imager.

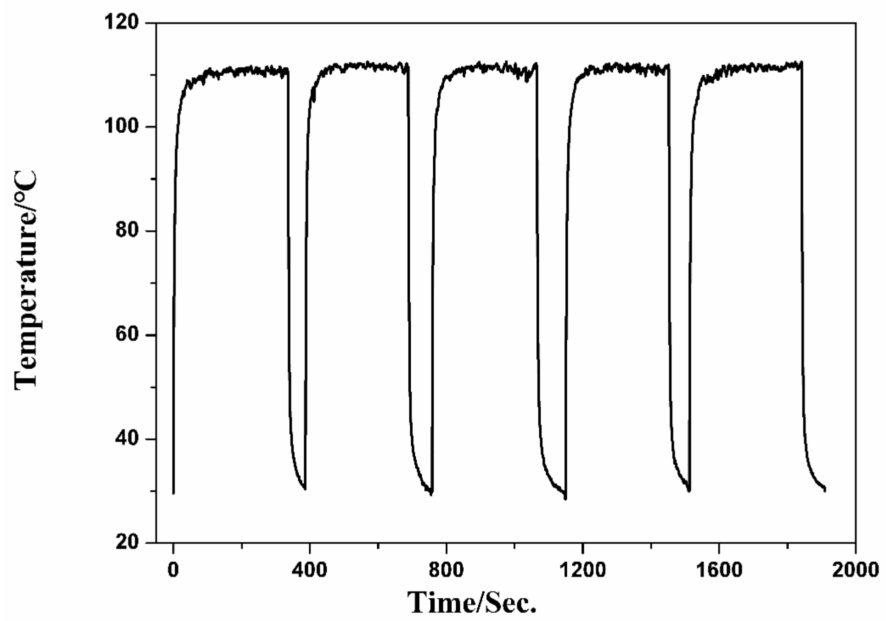

Figure S16. Photothermal cycling curve of the **Co-NDC** at 1.25 W/cm^2 irradiation by1064 nm laser.

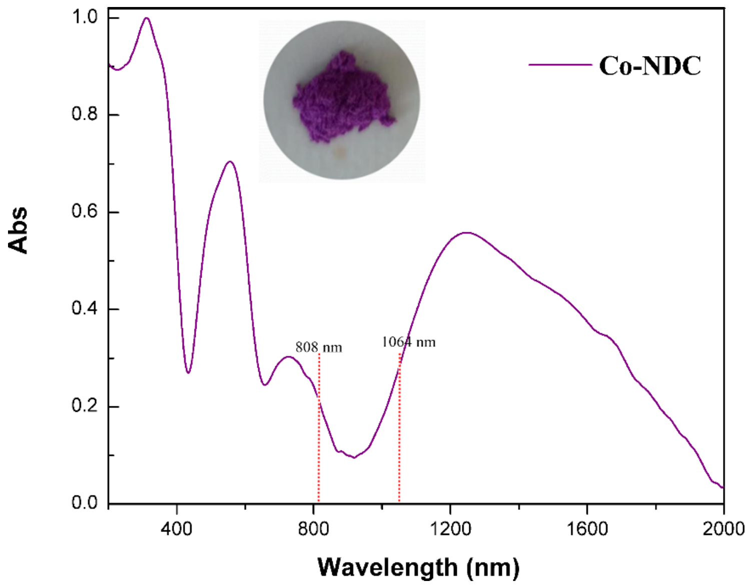

Figure S17. Vis spectra of **Co-NDC** in solid state measured at room temperature. Inset is the photograph of the as-made **Co-NDC**.

Table S1. Summary of the photothermal conversion efficiency of various MOF involved photothermal agents.

UV light source				
Samples	Light Intensity	Temperature ranges	ΔT	Ref
HKUST-1	0.5 W·cm^{-2}	25.0-124.7	99.3 °C in 30 min	S2
IR-MOF-3	0.5 W·cm^{-2}	26.4-118.6	92.2 °C in 30 min	
ZIF-8	0.5 W·cm^{-2}	26.1-70.5	44.4 °C in 30 min	
UiO-66	0.5 W·cm^{-2}	25.5-57.3	31.8 °C in 30 min	
MIL-101-NH_2- (Al)	0.5 W·cm^{-2}	~24-72 °C	48 °C in 30 min	S3
800-808 nm laser light source				
Zr-PDI$^{\bullet-}$ film	0.7 W·cm^{-2}	25-114 °C	89 °C in 200 s	S4
Ag-2D-CP	0.5 W·cm^{-2}	-	24.5 °C in 3 min	S5
La-MV-MOF (crystals)	2 W·cm^{-2}	23-111 °C	88 °C in 10 s	S6
La-MV-MOF (film)	2 W·cm^{-2}	23-145 °C	122 °C in 200 s	
Dy-*m*-TTFTB	0.7 W·cm^{-2}	22.8-90.1 °C	67.3 °C in 90 s	S7
Dy-*m*-TTFTB	0.7 W·cm^{-2}	23.0-82.3 °C	59.3 °C in 15 s	
Zn-MOF powder	0.4 W·cm^{-2}	25-255 °C	230 °C in 24 s	S8
Au@MOF	0.8 W·cm^{-2}	26.5-81.6 °C	55.1 in 10 min	S9
Fe-NDC	0.25 W·cm^{-2}	22.5-50 °C	27.5 °C in 10 s	This work
	0.5 W·cm^{-2}	22.5-67.6 °C	45.1 °C in 10 s	
	0.75 W·cm^{-2}	22.5-88.2 °C	65.7 °C in 10 s	
	1.0 W·cm^{-2}	22.5-108.7 °C	86.2 °C in 10 s	
980 and 1064 nm laser light				
Au@MOF (1064 nm)	1.8 W·cm^{-2}	26.5-100 °C	73.5 °C in 10 min	S9
Ru-MOF (980 nm)	-	26-51.7 °C	25.7 °C in 16 min	S10
1064 nm laser light				
Fe-NDC	0.25 W·cm^{-2}	25-49 °C	24 °C in 10 s	This work
	0.5 W·cm^{-2}	25-68 °C	43 °C in 10 s	
	0.75 W·cm^{-2}	25-90.1 °C	65.1 °C in 10 s	
	1.0 W·cm^{-2}	25-110 °C	85 °C in 10 s	
	1.25 W·cm^{-2}	25-135 °C	110 °C in 10 s	

References:

[S1] T. Yamashita, P. Hayes, Analysis of XPS spectra of Fe^{2+} and Fe^{3+} ions in oxide materials, *Appl. Surf. Sci.* **2008**, *254*, 2441–2449.

[S2] Espín, J.; Garzón-Tovar, L.; Carné-Sánchez, A.; Imaz, I.; Maspoch. D.; Photothermal Activation of Metal–Organic Frameworks Using a UV–Vis Light Source. *ACS Appl. Mater. Interfaces* **2018**, *10*, 9555–9562.

[S3] Espín, J.; Garzón-Tovar, L.; Boix, G.; Imaz, I.; Maspoch, D. The photothermal effect in MOFs: covalent post-synthetic modification of MOFs mediated by UV-Vis light under solvent-free conditions. *Chem. Commun.* **2018**, *54*, 4184-4187.

[S4] Lü, B.; Chen, Y.; Li, P.; Wang, B.; Müllen, K.; Yin, M. Stable radical anions generated from a porous perylenediimide metal-organic framework for boosting near-infrared photothermal conversion. *Nat. Commun.* **2019**, *10*, 767.

[S5] Li, M.-Q.; Zhao, M.; Bi, L.-Y.; Hu, Y.-Q.; Gou, G.; Li, J.; Zheng, Y.-Z. Two-Dimensional Silver(I)-Dithiocarboxylate Coordination Polymer Exhibiting Strong Near-Infrared Photothermal Effect. *Inorg. Chem.* **2019**, *58*, 6601−6608.

[S6] Wang, S.; Li, S.; Xiong, J.; Lin, Z.; Wei, W.; Xu, Y. Near-infrared photothermal conversion of stable radicals photoinduced from a viologen-based coordination polymer. *Chem. Commun.* **2020**, *56*, 7399-7402.

[S7] Su, J.; Cai, P.; Yan, T.; Yang, Z.-M.; Yuan, S.; Zuo, J.-L.; Zhou, H.-C. Enhancing the photothermal conversion of tetrathiafulvalene-based MOFs by redox doping and plasmon resonance. *Chem. Sci.* **2022**, *13*, 1657–1664.

[S8] Yan, T.; Li, Y.-Y.; Gu, Q.-Y.; Li, J.; Su, J.; Wang, H.-Y.; Zuo, J.-L. A Tetrathiafulvalene/Naphthalene Diimide-Containing Metal–Organic Framework with fsc Topology for Highly Efficient Near-Infrared Photothermal Conversion. *Inorg. Chem.* **2022**, *61*, 3078−3085.

[S9] Deng, X.; Liang, S.; Cai, X.; Huang, S.; Cheng, Z.; Shi, Y.; Pang, M.; Ma, P.; Lin, J. Yolk-Shell Structured Au Nanostar@Metal-Organic Framework for Synergistic Chemo-photothermal Therapy in the Second Near-Infrared Window. *Nano Lett.* **2019,** *19*, 6772-6780.

[S10] Zhang, T.; Cao, J.-W.; Jiang, X.; Chen, J.; Wang, T.; Chen, K.-J. Band Gap Modulation Enabled by TCNQ Loading in a Ru-Based Metal–Organic Framework for Enhanced Near-Infrared Absorption and Photothermal Conversion. *Cryst. Growth Des.* **2021,** *21*, 729-734.
